# Preoperative metabolic tumor volume of intrahepatic cholangiocarcinoma measured by ^18^F-FDG-PET is associated with the *KRAS* mutation status and prognosis

**DOI:** 10.1186/s12967-018-1475-x

**Published:** 2018-04-11

**Authors:** Yoshinobu Ikeno, Satoru Seo, Keiko Iwaisako, Tomoaki Yoh, Yuji Nakamoto, Hiroaki Fuji, Kojiro Taura, Hideaki Okajima, Toshimi Kaido, Shimon Sakaguchi, Shinji Uemoto

**Affiliations:** 1 Department of Surgery, Graduate School of Medicine, Kyoto University, 54 Kawahara-cho, Shogoin, Sakyo-ku, Kyoto, Japan; 2 Institute for Frontier Life and Medical Sciences, Kyoto University, Kyoto, Japan; 3 Diagnostic Radiology, Graduate School of Medicine, Kyoto University, Kyoto, Japan; 4 Department of Experimental Immunology, Immunology Frontier Research Center, Osaka University, Suita, Japan

**Keywords:** Intrahepatic cholangiocarcinoma, *KRAS* mutation, Glucose uptake, GLUT-1, ^18^F-FDG-PET

## Abstract

**Background:**

Surgical resection remains the mainstay of curative treatment for intrahepatic cholangiocarcinoma (ICC). Prognosis after surgery is unsatisfactory despite improvements in treatment and post-operative clinical management. Despite developments in the molecular profiling of ICC, the preoperative prediction of prognosis remains a challenge. This study aimed to identify clinical prognostic indicators by investigating the molecular profiles of ICC and evaluating the preoperative imaging data of ^18^F-fluorodeoxyglucose positron emission tomography (^18^F-FDG-PET).

**Methods:**

A retrospective analysis was performed on 50 consecutive patients with ICC who underwent curative hepatectomy after ^18^F-FDG-PET examination. To evaluate the molecular profiles of ICC, *KRAS* mutation status was assessed in resected specimens. For the assessment of glucose uptake, we observed the expression of glucose transporter-1 (GLUT-1) by immunohistochemistry. The data of ^18^F-FDG-PET were re-evaluated as follows: maximum standardized uptake value, metabolic tumor volume (MTV), and total lesion glycolysis (TLG). Cut-off values were determined using receiver operating characteristic (ROC) curve analysis. Cumulative overall survival (OS) was analyzed using the Kaplan–Meier analysis.

**Results:**

Overall, 16 (32.0%) patients had mutations in *KRAS*. Patients with mutated *KRAS* exhibited shorter OS than those with wild-type *KRAS* (5-year OS, 0% vs. 35.1%, *P *< 0.001). GLUT-1 expression was significantly higher in tumors with mutated *KRAS* than in tumors with wild-type *KRAS* (median, 4.0 vs. 1.0, *P* < 0.001). Survival was significantly different when stratified by expression of GLUT-1 (5-year OS, 0% vs. 46.5%, *P *<0.001). Among the ^18^F-FDG-PET parameters, the MTV and TLG were significantly higher in the mutated *KRAS* group than in the wild-type *KRAS* group (*P* = 0.013 and *P* = 0.026, respectively). ROC curve analysis revealed a cut-off value of 38 for the MTV, with the highest accuracy (area under the curve = 0.789; 95% confidence interval, 0.581–0.902) for predicting *KRAS* mutation. This cut-off value permitted stratification of OS (high vs. low: 5-year OS, 13.1% vs. 36.7%, *P* = 0.008).

**Conclusions:**

High MTV is associated with *KRAS* mutation and poor postoperative outcomes in patients with ICC, suggesting that the MTV of ICC measured by ^18^F-FDG-PET may provide useful information for tumor molecular profiles and prognosis.

**Electronic supplementary material:**

The online version of this article (10.1186/s12967-018-1475-x) contains supplementary material, which is available to authorized users.

## Background

Intrahepatic cholangiocarcinoma (ICC) is the second most common primary liver malignancy, with an increasing incidence and mortality worldwide [1–3]. Currently, surgical resection represents the curative treatment option, but surgery is only possible for selected patients. Even with optimal surgery, the 5-year overall survival (OS) rate is 15–40% [4, 5]. The incidence of post-surgical recurrence is 50–60% [3, 4], and recurrence is associated with a poor prognosis. It is thus essential to develop a multidisciplinary strategy for ICC to improve prognosis [6–9].

Recently, new therapies based on the molecular or genetic characteristics of ICC have been developed. In theory, it should be possible to select the appropriate therapy for each patient depending on the molecular or genetic characteristics of their individual tumors. Without surgical resection, however, there is little biological information in ICC, so it is difficult to select treatment before surgery.

In the era of genetic landscape and precision medicine, increasing evidence suggests that genetic mutation profiles allow the evaluation of prognosis of cancer postoperatively [10–14]. ICC has a relatively large number of actionable mutations compared to other gastrointestinal carcinomas. Several studies have demonstrated that *KRAS* mutation in ICC could affect prognosis [12–14]. Therefore, assessment of *KRAS* mutation status may contribute to the development of treatment strategies. A surrogate marker for *KRAS* mutation would provide genomic information without the need for biopsy or surgery. To identify this surrogate marker, it may be helpful to assess the biological effects of *KRAS* mutation.

Investigators have reported that tumor cells with *KRAS* mutation exhibit enhanced glucose uptake and glycolysis to survive in severe conditions (i.e., low-glucose and hypoxia) [15–17]. Glucose transporter-1 (GLUT-1) is a major glucose transporter in cholangiocarcinoma, and its expression is correlated with higher malignant potential in ICC [18, 19]. Positron emission tomography (PET) with ^18^F-fluorodeoxyglucose (^18^F-FDG), a glucose analog, is a less invasive modality that determines the glucose metabolism potential of tumors by quantifying ^18^F-FDG uptake. However, to date, the association between *KRAS* mutation and ^18^F-FDG-PET has not been reported in ICC.

We investigated the presence of *KRAS* mutation, the expression of GLUT-1, and ^18^F-FDG-PET parameters in 50 ICC patients, and examined whether there was an association between ICC prognosis and these factors.

## Methods

### Patients

The study was performed as a retrospective review of patients with mass-forming ICC who underwent hepatectomy at the Department of Surgery, Kyoto University Hospital (Kyoto, Japan) between May 2009 and August 2016. Inclusion criteria in the present study were: (1) diagnosis of ICC pathologically confirmed by two experienced pathologists, and (2) patients who underwent hepatectomy within 2 weeks of ^18^F-FDG-PET analysis. ICC was defined as a tumor developing from the intrahepatic bile duct at the secondary or more distal branches. Exclusion criteria were: (1) patients with apparent distant metastasis detected on preoperative imaging, and (2) patients with histologically different diseases. The clinicopathological characteristics and survival data of these patients were extracted from a prospectively maintained institutional database. Tumor stages were assessed according to the American Joint Committee on Cancer classification system, 7th edition [20]. Post-operative adjuvant chemotherapy was principally administered using gemcitabine for tumors in II–IV stages. Preoperative chemotherapy was not administered to patients in this study. Operative mortality was defined as death within 30 days of surgery; morbidity was evaluated according to the Clavien-Dindo classification system [21]. The latest survival data were collected on September 1, 2017. The study protocol was approved by the ethical committee of the Graduate School of Medicine, Kyoto University (G1019). Written informed consent was obtained from all study participants.

### *KRAS* mutational analysis

DNA extraction and mutation detection were performed using a modified protocol, as described previously [22, 23]. Briefly, DNA was extracted from formalin-fixed, paraffin-embedded (FFPE) tumor tissue sections by the QIAamp FFPE Tissue Kit (Qiagen, Venlo, Netherlands). Following DNA extraction, an assay with RASKET KIT (MBL, Nagoya, Japan) was performed according to the manufacturer’s protocol. We simultaneously examined 146 *KRAS* mutations located in exon 2 codons 12 and 13, exon 3 codons 59 and 61, and exon 4 codon 117.

### GLUT-1 expression analysis

Immunohistochemical staining was performed on 4-μm-thick FFPE sections as previously described, using GLUT-1 antibody (#ab15309, Abcam, Tokyo, Japan) diluted at 1:200 [24]. Membrane-predominant staining was regarded as positive. The grade of GLUT-1 expression was semi-quantitatively assessed according to the following scoring scheme: the percentages of tumor cells with strong staining were calculated in 10 fields (magnification 200×), and the mean percentage of stained tumor cells was calculated and scored on a 5-point scale (0 = 0%, 1 = 1–25%, 2 = 26–50%, 3 = 51–75%, and 4 = 76–100%) [24]. The evaluation of immunohistochemical analysis was performed by two independent, experienced researchers who were blinded to the clinicopathological data. Using the median value of immunostaining grade as the cut-off, patients were classified into high and low GLUT-1 expression groups.

## ^18^F-FDG-PET study and image analysis

^18^F-FDG-PET studies were performed using a PET/computed tomography (CT) scanner (Discovery ST Elite or Discovery IQ; GE Healthcare, Milwaukee, WI, USA). Patients fasted for at least 4 h before undergoing ^18^F-FDG-PET. The plasma glucose level was checked before the injection of ^18^F-FDG, and there were no patients with blood glucose level > 150 mg/dL in this study. Data acquisition started approximately 60 min after intravenous administration of ^18^F-FDG (injected dose: approximately 3.7 MBq/kg body weight). Initially, starting at the level of the upper thigh, low-dose CT scans were obtained with the following parameters: 40–60 mA, 120 kV, 0.6-s tube rotation, and 3.75-mm section thickness. The CT images were acquired during shallow breathing, and scanning included the area from the upper thigh to the skull. Immediately after the CT scans were acquired, PET emission scanning was performed with an acquisition time of 2–3 min per bed position. Whole-body PET images were attenuation-corrected using CT data and reconstructed using a 3D ordered-subsets expectation–maximization algorithm called VUE Point Plus (Discovery ST Elite: 14 subsets, two iterations, a matrix size of 128 × 128, a voxel size of 4.7 × 4.7 × 3.3 mm, and post-filtering at 5-mm full width at half maximum; Discovery IQ: 12 subsets, four iterations, a matrix size of 192 × 192, a voxel size of 3.3 × 3.3 × 3.3 mm, and post-filtering at 5-mm full width at half maximum). For quantitative analysis, at least two board-certified radiologists/nuclear medicine physicians assessed ^18^F-FDG accumulation on a workstation (Advantage Workstation 4.6; GE Healthcare) by calculating the standardized uptake value (SUV) in the regions of interest placed over the suspected lesions using all available clinical information and correlative conventional imaging for anatomic guidance. The SUV was calculated for the quantitative analysis of tumor ^18^F-FDG uptake as follows: SUV = C (kBq/mL)/ID (kBq)/body weight (kg), where C is the tissue activity concentration measured by PET and ID is the injected dose.

^18^F-FDG uptake was also quantitatively assessed by SUVs calculated in volumes of interest (VOIs) that were placed over regions of abnormal ^18^F-FDG uptake. The boundaries of each VOI were checked by comparison with fused CT to exclude adjacent ^18^F-FDG avid structures. The maximum SUV (SUV_max_) within the VOI was recorded for the primary tumor. Metabolic tumor volume (MTV) was defined as the total tumor volume segmented via the threshold SUV. The threshold of the mediastinal blood pool activity was used to define the lesions. For the threshold SUV established using mediastinal blood pool activity, a VOI of more than 5 × 5 × 5 voxels was drawn manually at the aortic arch. The average SUV at the aortic arch plus two standard deviations of the VOI was adopted as the threshold SUV for the tumor using the mediastinal blood pool. Total lesion glycolysis (TLG) was determined as a product of the average SUV (SUV_mean_) segmented via the threshold SUV multiplied by the number of voxels in the MTV (i.e., SUV_mean_ × MTV). For each patient, we defined the MTV and TLG as the sum of the MTVs and TLGs of all lesions, respectively.

### Statistical analysis

Continuous values are expressed as median (range) and were compared using the Mann–Whitney *U* test. Categorical variables were compared using Fisher’s exact test. The prognostic values of clinicopathological factors for survival were assessed using a Cox proportional hazard regression model for univariate and multivariate analyses. Hazard ratios with Wald 95% confidence intervals (CIs) were provided for the Cox regression models. OS was calculated from the date of surgery to the date of death or last follow-up according to the Kaplan–Meier method and analyzed by the log-rank test. Cut-off values of ^18^F-FDG-PET parameters to discriminate *KRAS* mutation status were determined by receiver operating characteristic (ROC) curve analysis. The optimal cut-off values, sensitivities, and specificities of ^18^F-FDG-PET parameters were determined using the Youden index. All analyses were two-sided, and differences were considered significant when *P* was < 0.05. All statistical analyses were performed using the JMP statistical software package (SAS Institute Inc., Cary, NC, USA).

## Results

### Patient characteristics

**Fig. 1:**
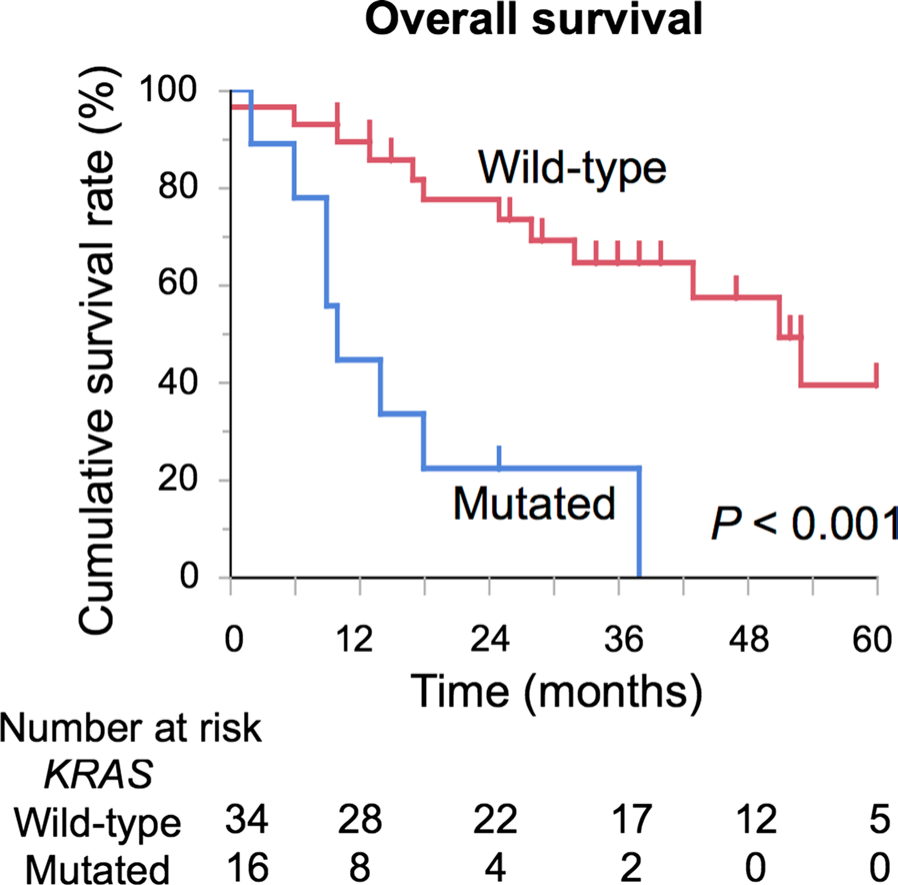
Survival of patients after curative surgery according to *KRAS* mutation. The Kaplan–Meier method was used to determine patient survival, and the log-rank test was used to compare survival between intrahepatic cholangiocarcinoma patients with wild-type and mutant *KRAS*

The clinicopathologic features of the patients are summarized in Table 1. No patients received any treatment before operation, and postoperative adjuvant chemotherapy was performed in 30 patients (60.0%). The overall morbidity rate was 36.0% (n = 18), and the class III/VI morbidity rate was 24.0% (n = 12). Follow-up was available in all cases and ranged from 9.0 to 91.0 months (median, 32.0 months). The median survival time was 22.5 months, with 3- and 5-year survival rates of 32.6 and 8.7%, respectively. Overall, 16 (32.0%) patients had mutations in *KRAS*. In multivariate analysis, *KRAS* mutation was an independent prognostic factor for OS (Additional file 1: Table S1). Furthermore, patients with mutated *KRAS* exhibited impaired OS compared with those with wild-type *KRAS* (5-year survival, 0% vs. 35.1%, *P *< 0.001, Fig. 1).

**Table 1:** Patient characteristics

Variables	Patients (*n *= 50)
Clinical factors	
Sex, male/female	29/21
Age (years)	69 (32–84)
HBsAg, positive, n (%)	2 (4.0)
HCV Ab, positive, n (%)	7 (14)
Child–Pugh class, A/B	48/2
CEA (ng/mL)	3.3 (0.4–133.1)
CA 19-9 (IU/mL)	199.8 (0.7–3055.0)
Tumor size (cm) (radiographical)	4.0 (1.0–14.0)
Treatment factors	
R0 resection, n (%)	48 (96)
Surgical procedures	
Extended/major/minor hepatectomy	22/22/6
Morbidity, C-D class III/IV, n (%)	12 (24)
Preoperative chemotherapy, n (%)	0
Adjuvant chemotherapy, present, n (%)	30 (60)
Pathological factors	
Tumor differentiation	
Well/moderate/poor	28/15/7
Vascular invasion, present, n (%)	34 (68)
Biliary invasion, present, n (%)	24 (48)
Lymph node metastasis, present, n (%)	15 (30)
Tumor number, multiple, n (%)	15 (30)
Tumor size (cm) (pathological)	3.5 (1.0–14.0)
AJCC stage, I/II/III/IV	2/10/13/25
*KRAS* mutation status	
Wild-type/mutated	34/16

*AJCC* American joint committee on cancer/international union against cancer classification, *CA 19*-*9* carbohydrate antigen 19-9, *CEA* carcinoembryonic antigen, *HBsAg* hepatitis B virus surface antigen, *HCV Ab* hepatitis C virus antibody, *R0 resection* no macroscopic and microscopic tumor remaining, *C*-*D* Clavien-Dindo classification system

### The association between *KRAS* mutation and clinicopathological factors

**Fig. 2:**
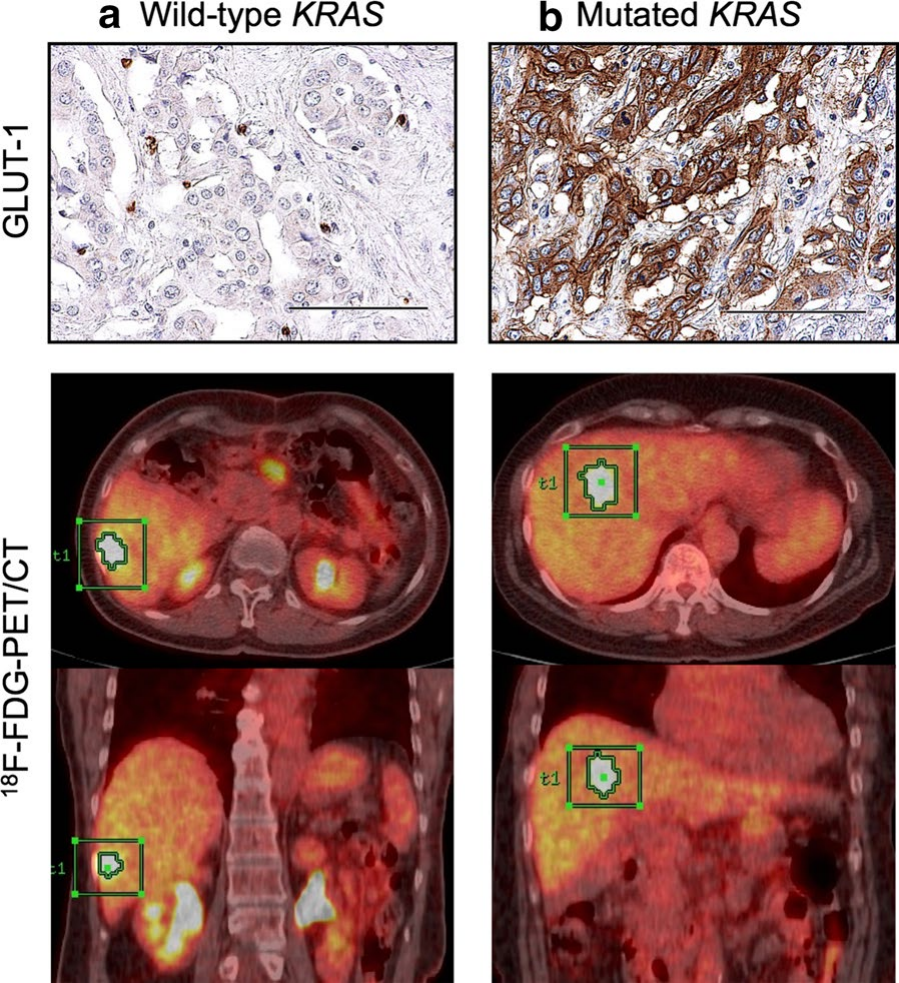
Immunohistochemical analysis for GLUT-1 expression in resected intrahepatic cholangiocarcinoma specimens and ^18^F-FDG-PET/CT scans. **a** A tumor with wild-type *KRAS* showed negative staining (score 0) for GLUT-1, and ^18^F-FDG-PET/CT scans showed modest accumulation of ^18^F-FDG in the tumor (MTV, 3.6; TLG, 31.9). **b** A tumor with mutated *KRAS* showed moderate membranous staining (score 3) for GLUT-1, and ^18^F-FDG-PET/CT scans showed intense accumulation of ^18^F-FDG in the tumor (MTV, 76; TLG 213). The scale bar represents 100 μm. *GLUT-1* glucose transporter-1, ^*18*^*F-FDG-PET* ^18^F-fluorodeoxyglucose positron emission tomography, *MTV* metabolic tumor volume, *TLG* total lesion glycolysis

**Fig. 3:**
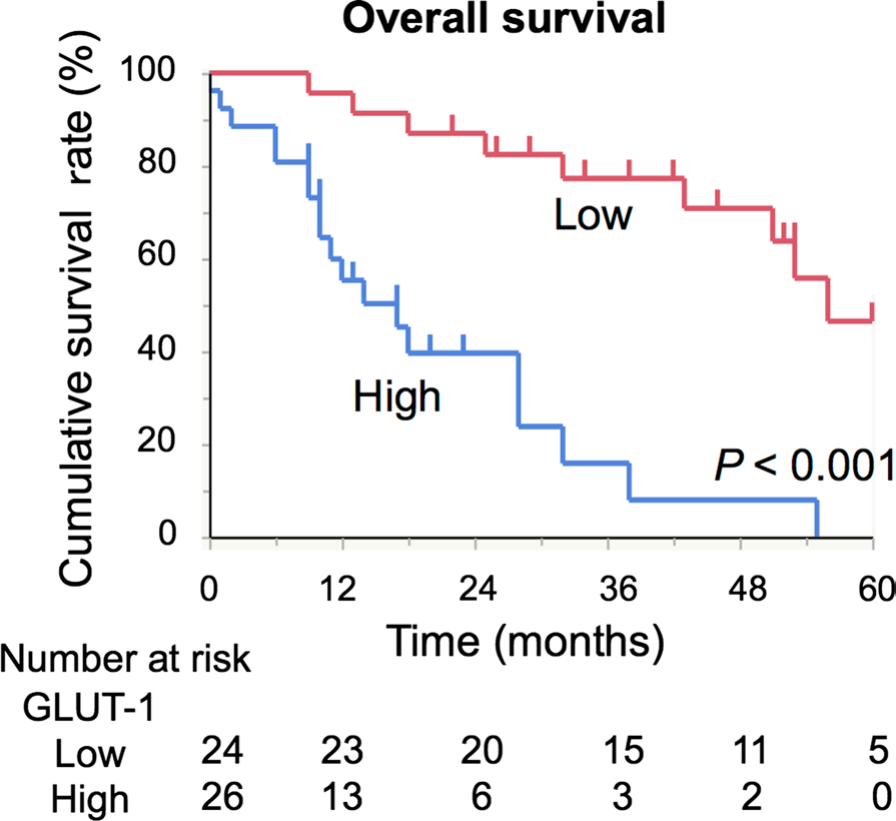
Survival of patients after curative surgery according to the grade of GLUT-1 expression. The Kaplan–Meier method was used to determine the patient survival and the log-rank test was used to compare survival of intrahepatic cholangiocarcinoma patients with high and low expression of GLUT-1. *GLUT-1* glucose transporter-1

Patients were divided into two groups based on *KRAS* mutation status (Table 2). There were no significant differences in clinical and treatment factors between the two groups. No correlation was observed between pathological factors, histologic type, vascular invasion, biliary invasion, lymph node metastasis, or tumor size or stage and *KRAS* mutation status. However, there were significant differences in intrahepatic tumor number (*P* = 0.008). To assess the impact of *KRAS* mutation on tumor glucose uptake, we examined the expression of GLUT-1 by immunohistochemistry in resected ICC specimens (Fig. 2). The GLUT-1 expression score was significantly higher in the mutated *KRAS* group than in the wild-type *KRAS* group (median, 4.0 and 1.0, respectively, *P *< 0.001). Furthermore, patients were divided into two groups according to expression of GLUT-1. High expression of GLUT-1 was detected in 26 of 50 ICC patients, and there were no significant differences in clinical or treatment characteristics between the two groups. However, patients with high expression of GLUT-1 had a higher SUV_max_ (*P* < 0.001), MTV (*P *= 0.008), and TLG (*P* < 0.001) of ^18^F-FDG-PET parameters compared to patients with low expression (Table 3). Survival after surgery was clearly divided when stratified by expression of GLUT-1 (5-year survival, 0% vs. 46.5%, *P *<0.001, Fig. 3).

**Table 2:** Comparative analysis of the clinicopathological findings between wild-type and mutated *KRAS* groups

Variables	*KRAS* Wild-type n = 34 (68.0%)	*KRAS* Mutated n = 16 (32.0%)	Univariate P*
Clinical factors			
Sex			
Male/female	19/15	10/6	0.763
Age (years)	69 (32 − 81)	69 (47 − 84)	0.303
CEA (ng/mL)	2.8 (0.4 − 133.1)	4.0 (1.0 − 116.6)	0.163
CA19-9 (IU/mL)	65.0 (0.8 − 3055.0)	38.7 (0.7 − 766.0)	0.593
Tumor size (cm)			
Radiographical	4.0 (1.0 − 13.0)	3.0 (1.0 − 14.0)	0.493
Treatment factors			
R0 resection, n (%)	33 (97.1)	15 (93.8)	0.542
Minor hepatectomy, n (%)	4 (11.8)	2 (12.5)	1.000
Morbidity			
C-D class III/IV, n (%)	9 (26.5)	3 (18.8)	0.728
Preoperative chemotherapy			
Present, n (%)	0	0	0
Adjuvant chemotherapy			
Present, n (%)	22 (64.7)	8 (50.0)	0.366
Pathological factors			
Tumor differentiation			
Well/moderate, n (%)	31 (91.2)	12 (75.0)	0.190
Poor, n (%)	3 (8.8)	4 (25.0)	
GLUT-1 expression	1.0 (0.0 − 4.0)	4.0 (2.0 − 4.0)	*< 0.001*
Vascular invasion			
Present, n (%)	22 (64.7)	12 (75.0)	0.533
Bile duct invasion			
Present, n (%)	16 (47.1)	8 (50.0)	1.000
Lymph node metastasis			
Present, n (%)	10 (29.4)	5 (31.3)	1.000
Tumor number			
Multiple, n (%)	6 (17.6)	9 (56.3)	*0.008*
Tumor size (cm)			
Pathological	3.6 (1.0 − 13.0)	3.4 (1.0 − 14.0)	0.532
AJCC stage			
IV, n (%)	16 (47.1)	9 (56.3)	0.762

*AJCC* American joint committee on cancer/international union against cancer classification, *CA 19*-*9* carbohydrate antigen 19-9, *CEA* carcinoembryonic antigen, *GLUT*-*1* glucose transporter-1, *R0 resection* no macroscopic and microscopic tumor remaining, *C*-*D* Clavien-Dindo classification system

*Statistically significant differences (*P *< 0.05) are shown in italic

**Table 3:** Comparative analysis of the clinicopathological factors according to the expression of GLUT-1

Variables	GLUT-1 Low n = 24 (48.0%)	GLUT-1 High n = 26 (52.0%)	Univariable P*
Clinical factors			
Sex			
Male/female	13/11	16/10	0.775
Age (years)	68 (32 − 83)	70 (46 − 81)	0.303
CEA (ng/mL)	2.8 (0.4 − 8.8)	3.6 (0.7 − 133.1)	0.265
CA19-9 (IU/mL)	38.7 (0.7 − 766)	65.5 (0.8 − 3055)	0.593
SUV_max_	4.5 (2.9 − 9.2)	7.0 (3.6 − 14.7)	*< 0.001*
MTV (cm^3^)	8.4 (1.5 − 604.0)	72.0 (3.6 − 777.0)	*0.008*
TLG (g)	20.2 (3.6 − 3201.2)	259.2 (27.0 − 3418.8)	*< 0.001*
Tumor size (cm)			
Radiographical	3.5 (1.0 − 11.0)	4.0 (1.0 − 14.0)	0.108
Treatment factors			
R0 resection, n (%)	23 (95.8)	25 (96.2)	1.000
Minor hepatectomy, n (%)	4 (16.7)	2 (7.7)	0.409
Morbidity			
C-D class III/IV, n (%)	5 (20.8)	7 (26.9)	0.745
Preoperative chemotherapy			
Present, n (%)	0	0	0
Adjuvant chemotherapy			
Present, n (%)	17 (70.8)	13 (50.0)	0.159
Pathological factors			
Tumor differentiation			
Well/moderate, n (%)	22 (91.7)	21 (80.8)	0.420
Poor, n (%)	2 (8.3)	5 (19.2)	
Vascular invasion			
Present, n (%)	18 (75.0)	16 (61.5)	0.372
Bile duct invasion			
Present, n (%)	11 (45.8)	13 (50.0)	0.785
Lymph node metastasis			
Present, n (%)	7 (29.2)	8 (30.8)	1.000
Tumor number			
Multiple, n (%)	2 (8.3)	13 (50.0)	*0.002*
Tumor size (cm) (pathological)	3.2 (1.0 − 10.8)	4.0 (1.2 − 14.0)	0.073
AJCC stage			
IV, n (%)	8 (33.3)	17 (65.4)	*0.047*

*AJCC* American joint committee on cancer/international union against cancer classification, *CA 19*-*9* carbohydrate antigen 19-9, *CEA* carcinoembryonic antigen, *MTV* metabolic tumor volume, *SUV*_*max*_ maximum standardized uptake value, *GLUT*-*1* glucose transporter-1, *R0 resection* no macroscopic and microscopic tumor remaining, *C*-*D*, Clavien-Dindo classification system

*Statistically significant differences (*P *< 0.05) are shown in italic

### The association of ^18^F-FDG-PET parameters with *KRAS* mutation status and prognosis

**Fig. 4:**
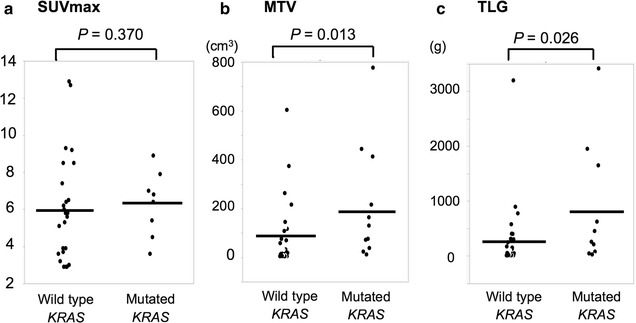
The association between *KRAS* mutation status and ^18^F-fluorodeoxyglucose positron emission tomography parameters. **a** Analysis of the maximum standardized uptake value (SUV_max_), **b** metabolic tumor volume (MTV), and **c** total lesion glycolysis (TLG) according to *KRAS* mutation status. Bars = means. Assessed using the Mann–Whitney *U* test

Next, we analyzed the association between *KRAS* mutation and ^18^F-FDG-PET parameters. There were no significant differences in SUV_max_ between the mutated *KRAS* group and the wild-type *KRAS* group (median, 5.7 and 5.8, respectively, *P *= 0.370, Fig. 4a). However, the MTV and TLG were significantly higher in the mutated *KRAS* group than in the wild-type *KRAS* group (median MTV, 75.9 vs 20.3, *P *= 0.013; median TLG, 259.1 vs 61.9, *P *= 0.026, Figs. 2, 4b, c).

**Fig. 5:**
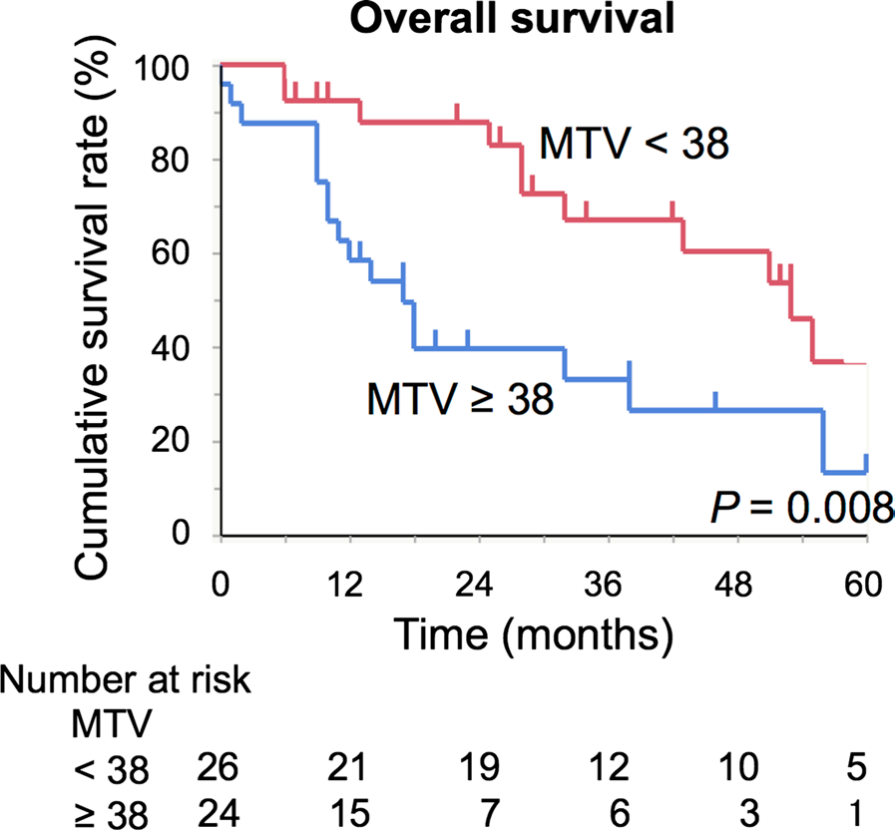
Overall survival of patients after curative surgery according to MTV. The Kaplan–Meier method was used to determine patient survival and the log-rank test was used to compare survival in patients with high and low MTV. *MTV* metabolic tumor volume

Finally, we examined the association between ^18^F-FDG-PET parameters and patient survival after surgery. To determine the optimal cutoff of the ^18^F-FDG-PET parameters, we performed a ROC curve analysis to discriminate *KRAS* mutation status (Additional file 2: Figure S1). This analysis showed the highest accuracy among the ^18^F-FDG-PET parameters, (area under the curve = 0.789; 95% CI 0.581–0.902) for an MTV cutoff value of 38. The sensitivity, specificity, and accuracy for predicting *KRAS* mutation status were 77.8, 67.9, and 68.0%, respectively. Patients with a high MTV (≥ 38) exhibited worse OS than those with a low MTV (< 38) (high vs. low: 5-year OS, 13.1% vs. 36.7%, *P* = 0.008, Fig. 5).

## Discussion

In this study, we investigated the *KRAS* mutation status in a cohort of 50 consecutive ICC patients who underwent radical hepatectomy and identified *KRAS* mutations in 32.0% of cases. The following features of our study are significant: (1) correlation between *KRAS* mutation and glucose uptake is recapitulated in ICC tumors, and (2) metabolic tumor volume on ^18^F-FDG-PET may provide useful information as a surrogate for prognosis, reflecting the impact of *KRAS* mutation on survival.

Malignant tumors can enhance tumor cell survival by genetic changes or modify glucose metabolism by cellular responses [25]. A number of genetic mutations in ICC have been identified [10–14]. In particular, *KRAS* mutation has been reported as a representative factor indicative of poor prognosis in ICC [12–14], and our patients with mutated *KRAS* showed significantly worse survival compared to those with wild-type *KRAS*. Regarding glucose metabolism, Warburg discovered that, even in the presence of oxygen, cancer cells undergo aerobic glycolysis rather than the normal oxidative phosphorylation. Aerobic glycolysis produces just two molecules of adenosine triphosphate (ATP) per molecule of glucose, while 36 ATP molecules are produced by oxidative phosphorylation. Cancer cells have an accelerated metabolism and increased requirements for ATP production. The reason why cancer cells, which need high ATP levels, take this inefficient pathway is not clear. To maintain high ATP levels for energy utilization, cancer cells may increase glucose transport through overexpression of GLUT-1. In this study, 52.0% of patients with ICC showed high expression of GLUT-1, which was associated with *KRAS* mutation. Whether the upregulation of GLUT-1 is attributable to *KRAS* mutation or whether GLUT-1 expression contributes to *KRAS* mutation remains to be established in ICC. High expression of GLUT-1 is associated with multiple tumors and tumor stage, and has been reported as a prognostic factor [26–28]. Patients with high GLUT-1-expressing tumors have a significantly poorer survival compared to patients with low GLUT-1 expression.

It has been reported that ^18^F-FDG accumulation reflects the *KRAS* mutational status of cancers [29–31]. We assessed three parameters measured by ^18^F-FDG-PET (SUV_max_, MTV, and TLG). In practice, SUV_max_ is the most commonly assessed parameter of ^18^F-FDG-PET, and previous reports have suggested that this parameter is associated with survival in patients with various cancers [32, 33]. Recently, several reports suggested that the volumetric parameters of tumors measured by ^18^F-FDG-PET, such as the MTV and TLG, are more accurate prognostic factors than SUV_max_ in patients with various malignancies [34, 35]. In this study, we have shown that *KRAS* mutations were significantly associated with high ^18^F-FDG uptake as calculated by the MTV and TLG, while SUV_max_ was comparable between the mutated *KRAS* group and wild-type group. Volumetric parameters measured by ^18^F-FDG-PET have advantages in terms of predicting *KRAS* mutation status. First, ^18^F-FDG-PET is non-invasive and harmless compared to performing a liver tumor biopsy. Second, volumetric parameters reflect the metabolic activity of the entire tumor mass in a three-dimensional manner. There was no association between SUV_max_ and *KRAS* mutation in this study, but this could be due to the intratumoral heterogeneity of the *KRAS* mutation status [13, 36]. SUV_max_ exhibits only the highest intensity of ^18^F-FDG uptake in the tumor and cannot reflect the metabolic activity of the entire tumor. Of the three parameters, the MTV and TLG were associated with *KRAS* mutation, and ROC analysis showed that the MTV was the best predictor of *KRAS* mutation.

In this study, the median SUV_max_ of the tumor lesions was 5.8 (range, 2.9–14.7). No patient with ICC had too little ^18^F-FDG uptake to detect the tumor lesion by FDG-PET despite of detectable tumor by CT or magnetic resonance imaging (MRI). When it was difficult to determine boundaries for obscure tumors, the tumor margin was identified using preoperative imaging, such as CT and/or MRI. Morphological size measured by CT or MRI was not significantly different between tumors with mutated *KRAS* and those with wild-type *KRAS*. In addition, it is well known that ICC tumors are frequently accompanied by central necrosis as they increase in size. Metabolic volume measured by ^18^F-FDG-PET more accurately reflects tumor viability than does radiographic volume, particularly as it takes into account tumor activity.

The current study has a limitation. It was a retrospective design conducted in a single institutional cohort of patients and involved a small study population of 50 consecutive ICC patients, including only 16 patients with *KRAS* mutation. This weakened the statistical power of our analysis. In addition, patient-selection bias might have influenced the statistical results. However, this study focused exclusively on ICC, rather than on biliary tract cancer, contributing to a better understating of *KRAS*-related molecular biology. Therefore, this should be considered a preliminary report, and further prospective studies with larger patient cohorts are required to validate the combination of ^18^F-FDG-PET parameters in association with somatic mutations and prognosis for patients with ICC.

## Conclusions

In this study, we demonstrated that *KRAS* mutation is associated with GLUT-1 expression and volumetric parameters of ^18^F-FDG-PET in ICC tumors. *KRAS* mutation affects the prognosis of ICC patients undergoing surgical resection and is associated with tumor glucose uptake. Moreover, our results suggest that ^18^F-FDG-PET may serve as a potential biomarker for *KRAS* mutation status and survival in ICC.

## Additional files

**Additional file 1: Table S1.** Univariate and Multivariate analyses of prognostic factors for overall survival.

**Additional file 2: Figure S1.** The ROC curve analysis of the performance of ^18^F-FDG-PET parameters for predicting *KRAS* mutation status. (a) Maximum standardized uptake value (SUVmax), (b) metabolic tumor volume (MTV), and (c) total lesion glycolysis (TLG). Note the high area under the ROC curve (AUC), 95% confidence interval (CI), and cutoff value (red font). *ROC* receiver operating characteristic, *AUC* area under the curve.

## Abbreviations

ICC: intrahepatic cholangiocarcinoma
GLUT-1: glucose transporter-1
^18^F-FDG-PET: ^18^F-fluorodeoxyglucose positron emission tomography
FFPE: formalin-fixed, paraffin-embedded
CT: computed tomography
MRI: magnetic resonance imaging
SUV_max_: maximum standardized uptake value
VOI: volume of interest
MTV: metabolic tumor volume
TLG: total lesion glycolysis
95% CIs: 95% confidence intervals
OS: overall survival
ROC: receiver operating characteristic
ATP: adenosine triphosphate
