# Supplementary material for: Preoperative metabolic tumor volume of intrahepatic cholangiocarcinoma measured by 18F-FDG-PET is associated with the KRAS mutation status and prognosis
Source: J Transl Med. 2018 Apr 11;16:95. doi: 10.1186/s12967-018-1475-x (PMC5896043; doi:10.1186/s12967-018-1475-x)
Supplement: Supplementary file 1 — Additional file 1: Table S1. Univariate and Multivariate analyses of prognostic factors for overall survival. [file 12967_2018_1475_MOESM1_ESM.docx]

**Table S1.** Univariate and Multivariate analyses of prognostic factors for overall survival

| **Variables** | Median |  | Univariate |  |  |  | Multivariate |  |
| --- | --- | --- | --- | --- | --- | --- | --- | --- |
| (Patient number) | (months) | HR | 95% CI | *P*＊ |  | HR | 95% CI | *P*＊＊ |
| ***Clinical factors*** |  |  |  |  |  |  |  |  |
| CEA (ng/mL) |  |  |  |  |  |  |  |  |
| < 4 (31) | 51 | 1 |  |  |  |  |  |  |
| ≥ 4 (19) | 28 | 1.78 | 0.76−4.08 | 0.174 |  | － |  |  |
| CA19-9 (U/mL) |  |  |  |  |  |  |  |  |
| < 200 (36) | 55 | 1 |  |  |  |  |  |  |
| ≥ 200 (14) | 14 | 4.59 | 2.10−10.2 | **0.001** |  | － |  |  |
| Surgical procedures |  |  |  |  |  |  |  |  |
| Major+minor (28) | 51 | 1 |  |  |  |  |  |  |
| Extended (22) | 25 | 2.17 | 0.98−5.26 | 0.054 |  | － |  |  |
| Adjuvant chemotherapy |  |  |  |  |  |  |  |  |
| Absent (20) | 17 | 1 |  |  |  |  |  |  |
| Present (30) | 38 | 0.72 | 0.33−1.60 | 0.407 |  | － |  |  |
| ***Pathological factors*** |  |  |  |  |  |  |  |  |
| Tumor differentiation |  |  |  |  |  |  |  |  |
| Well/Moderately (43) | 43 | 1 |  |  |  |  |  |  |
| Poorly (7) | 28 | 0.62 | 0.26−1.71 | 0.333 |  | － |  |  |
| Vascular invasion |  |  |  |  |  |  |  |  |
| Absent (16) | 56 | 1 |  |  |  |  |  |  |
| Present (34) | 28 | 2.17 | 0.89−6.50 | 0.091 |  | － |  |  |
| Biliary invasion |  |  |  |  |  |  |  |  |
| Absent (26) | 56 | 1 |  |  |  |  |  |  |
| Present (24) | 25 | 2.69 | 1.24−6.30 | **0.012** |  | － |  |  |
| Lymph node metastasis |  |  |  |  |  |  |  |  |
| Absent (35) | 51 | 1 |  |  |  |  |  |  |
| Present (15) | 32 | 1.64 | 0.73−3.53 | 0.221 |  | － |  |  |
| Tumor number |  |  |  |  |  |  |  |  |
| Solitary (35) | 53 | 1 |  |  |  | 1 |  |  |
| Multiple (15) | 17 | 3.81 | 1.59−9.17 | **0.003** |  | 1.57 | 0.56−4.49 | 0.394 |
| Tumor size (cm) |  |  |  |  |  |  |  |  |
| < 4 (32) | 43 | 1 |  |  |  |  |  |  |
| ≥ 4 (18) | 28 | 1.42 | 0.63−3.12 | 0.394 |  | － |  |  |
| AJCC stage |  |  |  |  |  |  |  |  |
| I+II+III (22) | 56 | 1 |  |  |  | 1 |  |  |
| IV (20) | 28 | 2.92 | 1.30−7.03 | **0.009** |  | 2.18 | 0.92−5.49 | 0.077 |
| *KRAS* mutation status |  |  |  |  |  |  |  |  |
| Wild-type (34) | 53 | 1 |  |  |  | 1 |  |  |
| Mutated (16) | 13 | 5.21 | 2.20−12.3 | **< 0.001** |  | 3.45 | 1.23−9.62 | **0.018** |
| **Abbreviations**: *AJCC*, American Joint Committee on Cancer/International Union Against Cancer Classification; *CA 19-9,* carbohydrate antigen 19-9; *CEA,* carcinoembryonic antigen; *HR*, hazard ratio; *CI,* confidence interval. | | | | | | | | |

＊Assessed by Cox hazard model. ＊＊Factors with *P*<0.05 in univariate analysis were entered into a multivariate stepwise regression model. Statistically significant differences (*P*<0.05) are shown in bold.
