# Supplementary figures and images for: Preoperative metabolic tumor volume of intrahepatic cholangiocarcinoma measured by 18F-FDG-PET is associated with the KRAS mutation status and prognosis
Source: J Transl Med. 2018 Apr 11;16:95. doi: 10.1186/s12967-018-1475-x (PMC5896043; doi:10.1186/s12967-018-1475-x)

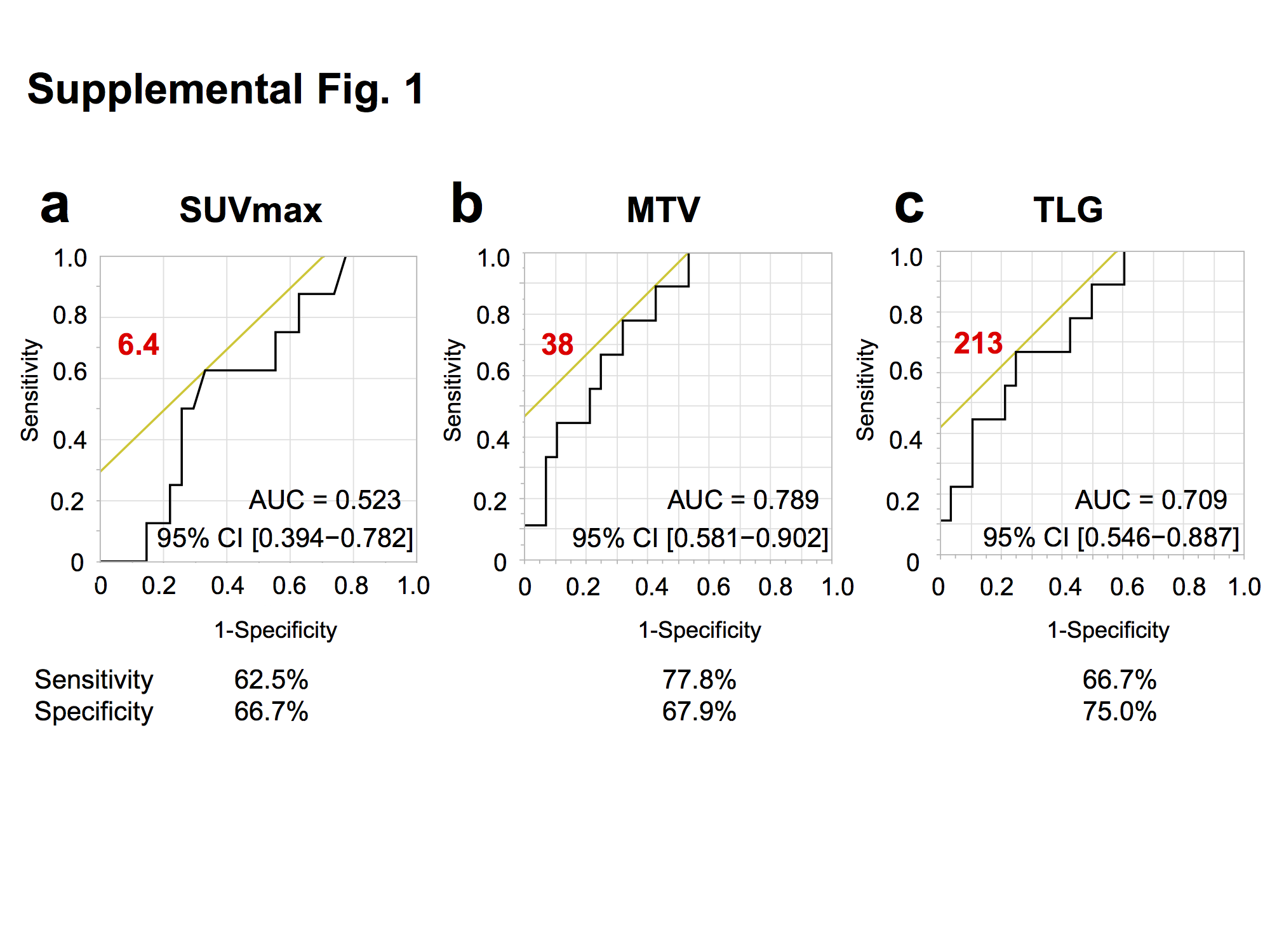

Supplement: Supplementary file 2 — Additional file 2: Figure S1. The ROC curve analysis of the performance of 18F-FDG-PET parameters for predicting KRAS mutation status. (a) Maximum standardized uptake value (SUVmax), (b) metabolic tumor volume (MTV), and (c) total lesion glycolysis (TLG). Note the high area under the ROC curve (AUC), 95% confidence interval (CI), and cutoff value (red font). ROC receiver operating characteristic, AUC area under the curve. [file 12967_2018_1475_MOESM2_ESM.tiff]
